# Unraveling the effects of the Ebola experience on behavior choices during COVID-19 in Liberia: a mixed-methods study across successive outbreaks

**DOI:** 10.1186/s44263-024-00054-5

**Published:** 2024-04-01

**Authors:** Laura A. Skrip, Malcom B. Weller, Sheikh Dukuly, Neima Candy, Wahdae-Mai Harmon-Gray, Adolphus Clarke, Bernice T. Dahn

**Affiliations:** 1 School of Public Health, College of Health Sciences, University of Liberia, 1000-10 Monrovia, Liberia; 2 Expanded Program on Immunization Unit, Ministry of Health, Monrovia, Liberia; 3 College of Health Sciences, University of Liberia, Monrovia, Liberia

**Keywords:** SARS-CoV-2, Liberia, Behavior during outbreaks, Ebola outbreak, Pandemics, Vaccination behavior, Social and economic consequences

## Abstract

**Background:**

The burden of the COVID-19 pandemic in terms of morbidity and mortality differentially affected populations. Between and within populations, behavior change was likewise heterogeneous. Factors influencing precautionary behavior adoption during COVID-19 have been associated with multidimensional aspects of risk perception; however, the influence of lived experiences during other recent outbreaks on behavior change during COVID-19 has been less studied.

**Methods:**

To consider how the direct disease experience (“near misses”) and behavior change during the 2014–2016 Ebola virus disease (EVD) outbreak may have impacted behavior change during the early waves of the COVID-19 outbreak in West Africa, we analyzed data from a mixed-methods study that included a phone-based survey and in-depth interviews among vaccinated Liberian adults. Logistic regression via generalized estimating equations with quasi-likelihood information criterion (QIC)-based model selection was conducted to evaluate the influence of the interaction between and individual effects of the outbreak (EVD and COVID-19) and the “near-miss” experience on adoption of individual precautionary behaviors. Thematic analysis of interview transcripts explored reasons for differential behavior adoption between the two outbreaks.

**Results:**

At the population level, being a “near miss” was not associated with significantly different behavior during COVID-19 versus Ebola; however, overall, people had lower odds of adopting precautionary behaviors during COVID-19 relative to during Ebola. Participants who report near miss experiences during Ebola were significantly more likely to report having a household member test positive for COVID-19 (p<0.001). Qualitatively, participants often reflected on themes around more proximal and personal experiences with Ebola than with COVID-19; they also commented on how EVD led to better preparedness at the systems level and within communities for how to behave during an outbreak, despite such awareness not necessarily translating into action during COVID-19.

**Conclusions:**

The results suggest that perceived proximity and intensity to disease threats in space and time affect behavioral decisions. For successive disease threats, comparisons of the present outbreak to past outbreaks compound those effects, regardless of whether individuals were directly impacted via a “near-miss” experience. Measures, such as risk communication and community engagement efforts, that gauge and reflect comparisons with previous outbreaks should be considered in response strategies to enhance the adoption of precautionary behavior.

**Supplementary Information:**

The online version contains supplementary material available at 10.1186/s44263-024-00054-5.

## Background

Across the world, the COVID-19 pandemic impacted populations with variable degrees of reported morbidity, mortality, and socioeconomic burden [1]. Countries in the Americas, Europe, and Asia tended to experience higher per capita rates of mortality and more severe disease than countries in Africa [2]. Countries in sub-Saharan Africa, specifically, reported some of the lowest case and death counts globally, yet the social and economic burden of the pandemic has been high [3]. Reflecting a complex interplay of climatic conditions, demography, surveillance capacity, and preexisting immunity [4–7], differential epidemiological patterns have also been attributed to differences in approaches to pandemic response [8]. While a set of “typical” containment measures were adopted by most countries [9–11], the success of efforts in controlling COVID-19 was related to how well such efforts recognized event-specific transmission patterns [12] and how well they were adapted to cultural, historical, and socioeconomic differences across countries and within countries [13, 14].

**Fig. 1:**
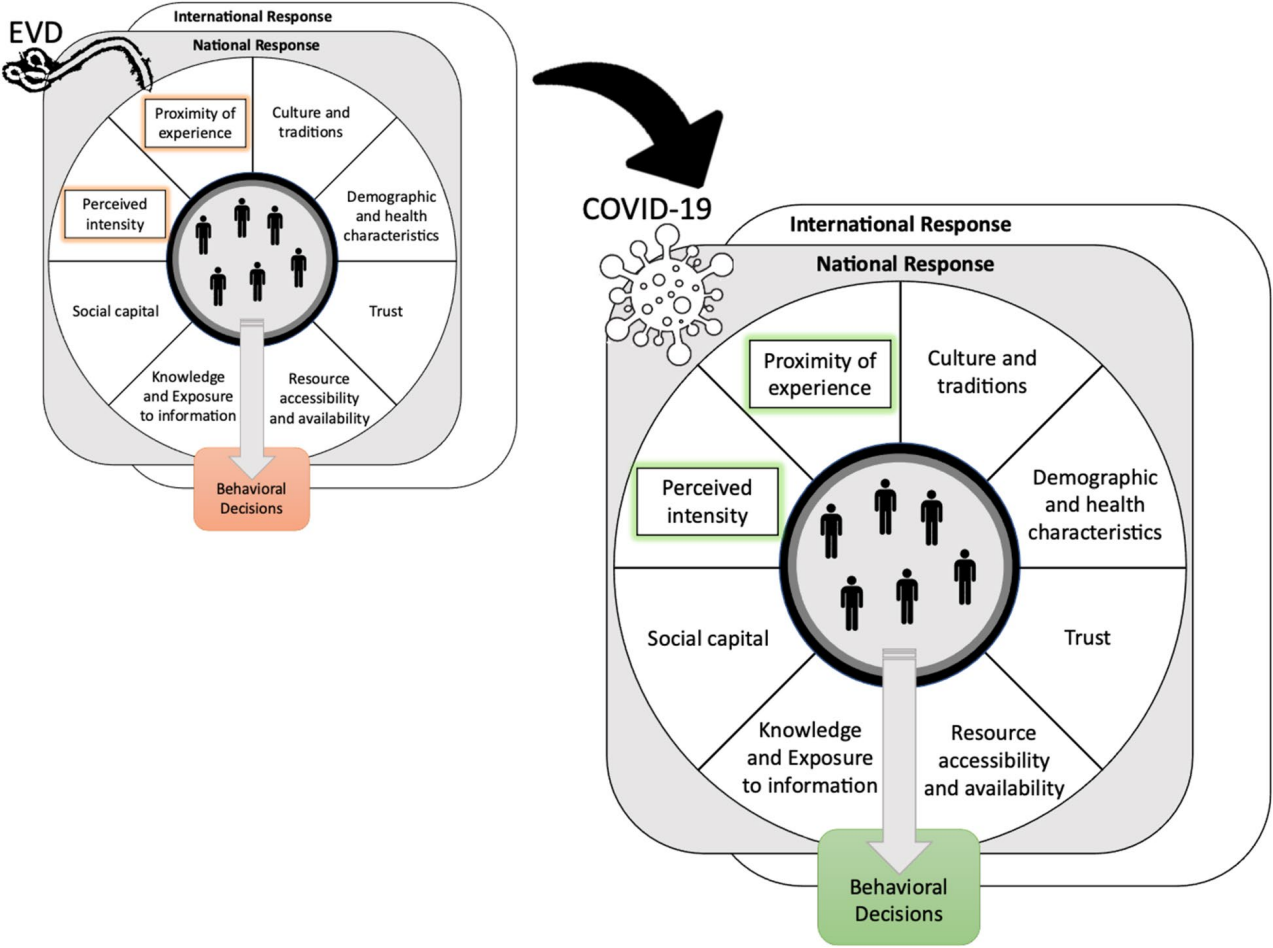
Role of successive outbreaks in modifying risk perception and behavior choices. Individual behavioral decisions occur under the influence of personal experiences and other individual-level factors within the context of community- and national-level factors such as local (e.g., community) rules, collective efficacy, and/or national policies that vary in stringency of implementation. The proximity and perceived severity or intensity (i.e., morbidity and mortality) of the situation are particularly influential factors in low-resource settings. For Liberia, decision-making around the adoption of precautionary behavior during COVID-19 was moderated by comparisons with the Ebola experience

In the context of national policies and guidance on COVID-19 response strategies and the variable stringency with which they were implemented [15], individuals ultimately decided whether or not to adhere [16, 17]. There is a diverse and growing body of literature on risk perception during COVID-19 [18–21] and factors associated with adherence to response recommendations [22–24]. For COVID-19 and other diseases, behavioral response has been associated, sometimes even counterintuitively in terms of the direction of the association, with perceptions of the intensity and proximity of the ongoing disease outbreak—such as being close to an epicenter or having a high risk of infection [21, 25–27]. Other factors, such as socioeconomic status, demographic characteristics, and physical and social needs, have been identified as modifying risk perception and decisions around health behavior [28–30]. The complex, multilevel influences on risk perception and behavior change during outbreaks have accounted for preexisting knowledge, perceptions, and protective measures for a given disease [31]. However, models of risk perception and health behavior tend not to capture the ways in which a recent history of other outbreaks may modify risk perception and behavior change in the context of an outbreak of a newly emerging disease. In particular, the feedback on how risk perception and corresponding behavior change influenced personal experiences with the disease in the past outbreak, such as whether people felt appropriately protected or had close encounters with the disease, could affect their current perceptions of risk and behavior change (Fig. 1).

In Liberia, the rollout of COVID-19 response measures took place within a post-Ebola context. The 2014–2016 Ebola outbreak in West Africa was associated with an unprecedented number of cases and deaths [32]. Like COVID-19 was a novel threat, Ebola had not previously affected West Africa. Its transmission via direct contact with infected bodily fluids was associated with high rates of spread in a context where (before intervention) essential infection prevention and control measures were often lacking during the practices of caring for the sick and burying the deceased [33]. Such practices, in combination with a high case fatality rate in the absence of effective, curative treatment options, had significant public health consequences. While transmission of COVID-19 via droplets was likely high in the context due to cultural and structural factors [34], reports of ultimately lower local case fatality and less frequent severity of illness in comparison to other settings contrasted with the Ebola experience.

The “back-to-back” outbreaks of Ebola and COVID-19 differed starkly in terms of the apparent morbidity and mortality; however, many response strategies were similar [35, 36]. While the systems-level response structures from Ebola [37] were quickly activated to address COVID-19, individual-level behavior varied, and heavily policed efforts were instituted to promote more adherence [38]. The reliance on top-down approaches across sub-Saharan Africa early in the COVID-19 pandemic and the disadvantages of the approach in that context have been recognized [36]. The intense stringency of enforcement faded quickly due to resource constraints and population reactions, with decisions on behavior change during the waves of COVID-19 in Liberia becoming more local at community and individual levels. In the process, different personal experiences with Ebola may have led to a spectrum of passive adaptation [39] during the waves of COVID-19 in the country. More direct experience with Ebola virus disease (EVD), such as due to having a household member experience Ebola infection [40], could be considered “near miss” or proximal experiences. In contrast, others who likely knew of cases in their communities or neighboring communities reflect more “remote miss” experiences. Near-miss behavioral dissonance has been discussed in the context of COVID-19 [41, 42], but limited empirical evidence exists.

Here, we consider successive outbreaks of diseases with differing epidemiology but similar approaches to control. We offer insight into the experience of COVID-19 in a post-Ebola context in general. We specifically investigate the practice of precautionary behaviors during COVID-19 versus during EVD for Liberians who reported “near misses” during the Ebola outbreak versus those who did not report “near misses.”

## Methods

### Study design and sampling

The present analysis used data from a mixed-methods study investigating the social, demographic, and clinical drivers of COVID-19 vaccination behavior in post-Ebola Liberia [43]. The study “Methods” and “Results” are presented according to the Strengthening the Reporting of Observational Studies in Epidemiology (STROBE) guideline (see Additional file 1).

The study population included all vaccinated adults (aged 18 and over) who were reflected in the Ministry of Health (MOH) database. This national database included individuals who received vaccines after the rollout of a digital system or who received vaccines prior to the digitalization of the process but whose information had been manually entered from the paper ledgers. Geographic differences in the rate of entry from paper ledgers and the availability of phone numbers may have biased the sample, as discussed further in the “Limitations” section of the “Discussion.”

A sample size of 1118 participants was targeted. To achieve 80% power at the 0.05 significance level within a population of 1,104,734 individuals (people partially or fully vaccinated as of January 14, 2022), data from a sample of size 1118 was estimated to be needed to detect with 1% margin of error a sample proportion of 3% experiencing the primary—and what was expected to be the least frequent—outcome: an adverse event following immunization (AEFI) (consistent with findings on the AstraZeneca vaccine available at the time of study planning [44]). The sample size was expected to be sufficient for analyses to address secondary objectives, including multivariable analyses with about 10 covariates, per the principle requiring approximately 10 outcome events (e.g., precautionary behavior) for each variable in the regression. It was also calculated as sufficient, given 80% power and 0.05 significance level, for detecting a moderate odds ratio of behavior adoption among those with “near-miss” experience with Ebola compared to those without an experience among vaccinated Liberians (requiring 816 participants).

The study team obtained the listing of all digitally documented vaccinated persons aged 18 and over from the Expanded Programme on Immunization (EPI) Unit at the MOH. The list included 213,020 unique persons who had been vaccinated (i.e., status not “scheduled”), who were Liberian, and who had an EPI-assigned vaccine ID. Cluster sampling at the county level, proportional to the county’s contribution to the overall vaccinated population at the time of sampling, was undertaken. In anticipation of high nonresponse rates, twice as many persons as would be needed to achieve the target sample size were randomly sampled using the proportional approach. The resulting list was returned to EPI for the data manager to manually add the phone numbers to the database, as this field could not be part of the initial data pull due to a limitation in the query builder. This initial list was quickly exhausted due to nonresponse or inaccurate contact numbers in the database, and the process was repeated two more times. After the first random sample, lists that were five times the target sample size were subsequently sampled and sent for contact numbers. When lists were returned, individuals without an available phone number were removed from the database, and potential participants with phone numbers were given study IDs and assigned to data collectors so they could initiate contact. Out of the 5794 unique persons sampled and sent to the EPI data manager, 3400 were returned with local contact numbers in the database and were assigned study IDs. From these, 1120 people were reached and agreed to participate.

For the in-depth interviews, while a randomly selected subset of 30 survey participants was invited, 25 were ultimately available and consented to participate. Random selection was made from separate lists of individuals who did not have adverse events and individuals who reported feeling adversely after immunization. Phone-based interviews via speaker phone were undertaken to enable the recording of responses.

### Data collection

Ahead of and during data collection, an intensive information campaign was conducted to share details about the phone survey to enhance buy-in from potential respondents when the study team called them. The Health Promotion Unit at the Liberia Ministry of Health partnered with the study team to develop and disseminate a short video post for social media and a radio jingle designed to dispel rumors or potential concerns around participation in the phone survey. The approach was undertaken to build credibility since those conducting the surveys could reference materials online and on the radio for people to view and learn about the study. It was also used to overcome barriers around the approach, as phone surveys are not common practice in Liberia, and the general population often considers calls from strangers to be scams.

For the phone surveys, data collectors made up to three attempts to contact potential participants at different times of the day and on weekdays and weekends to account for unavailability due to work schedules. An alternative was selected if a potential participant was not reached within the three attempts. Upon contact, a potential participant was informed how his/her number was obtained and given a short overview of the study. Verbal consent was requested and noted in a database.

The phone survey instrument consisted of primarily closed-ended questions that inquired about each participant’s sex, age, community of residence, reasons for vaccination, history of comorbidities (e.g., diabetes, hypertension), employment status, and total number of people in the household. In addition, participants were asked about the frequency of behaviors during EVD and during COVID-19 relative to before. For instance, they responded whether they washed their hands with more, the same, or less frequency during the COVID-19 outbreak in Liberia versus before it. Other behaviors that were interrogated included attendance at worship services, time in public places, use of public transportation, and visits to health facilities. Participants were also asked whether they had a family or household member who was infected during the Ebola outbreak and whether they had a family or household member test positive for COVID-19. Lastly, participants were asked about the likelihood of their acceptance of the prophylactic Ebola vaccine if it was made available. The survey tool is included in Additional file 2: Phone Survey Data Collection Tool.

As part of the in-depth interviews, participants were asked questions about their motivations to pursue vaccination against COVID-19, their experiences at the vaccination site and with any side effects after receiving the vaccine, and their behaviors to protect themselves from COVID-19. Interview participants were also asked, “tell me how you feel when you hear the word Ebola and why you feel that way.” They were then asked the same question but for COVID-19 instead of Ebola. The interview guide is included in Additional file 2: In-Depth Interview Data Collection Tool.

### Data analysis

Descriptive statistics were used to summarize sociodemographic and clinical characteristics, motivations for COVID-19 vaccination, acceptance of future vaccines within the sample overall, and by EVD near-miss status. Frequencies and percentages were used for categorical variables, and medians and interquartile ranges (IQRs) were calculated for continuous variables. Statistical differences in the characteristics between those reporting EVD near-miss experiences and those not reporting such experiences were evaluated using the chi-squared test or Fisher’s exact test and the Mann-Whitney *U*-test for categorical and continuous variables, respectively.

For each behavior, logistic regression analyses were performed using generalized estimating equations (GEEs) with robust variance estimates [45] to interrogate whether the practice of precautionary behavior (undertaking riskier behaviors less often or preventative behavior more often) was associated with outbreak and/or near-miss status while accounting for age, sex, geographic region, and education level of participants. Precautionary behaviors included increased hand-washing, decreased use of public transportation, decreased visits to places of worship, and decreased visits to health facilities. The GEE method was used to account for the longitudinal nature of the outcome variable or the adoption of precautionary behaviors at two points in time: during the Ebola outbreak and during the COVID-19 outbreak in Liberia. The method could address correlations in the outcome between timepoints (Ebola versus COVID-19) and within the same participant while allowing for inference at the population level of the effect of “near-miss” experience. The importance of the main effects of outbreak and near-miss status and their interaction in explaining the uptake of precautionary behaviors were explored and evaluated based on the QIC (quasi-likelihood information criterion). For model selection, the first step was to remove the interaction term and recalculate the QIC for comparison with the full model. With the interaction term still removed, each main effect was removed individually and then together, with the latter reflecting the most parsimonious model, including only the sociodemographic characteristics used for adjustment. Changes in QIC were documented for each iteration, and the model with the least QIC was selected for a given behavioral outcome. All analyses were performed using R statistical software, version 4.2.0.

For the in-depth interview data analysis, particularly responses to questions on perceptions around Ebola and perceptions of COVID-19, thematic analysis [46] involved three researchers independently coding the transcripts and then collaboratively reviewing and defining themes. Direct quotes from the transcripts are provided to support the themes. Qualitative data analysis was conducted using Atlas.ti Version 23.1.1.

## Results

### Overview of phone survey sample

Phone surveys were conducted with 1120 vaccinated adults from 13 counties in Liberia. The proportional sampling approach aimed at higher representation from counties with a higher proportion of the vaccinated population, namely Grand Bassa, Lofa, and Montserrado counties (Additional file 2: Fig. S1). However, the difference between the proposed proportion and the actual proportion of the sample from each county reflected the “underrepresentation” of some counties, such as Lofa and Maryland, and the “overrepresentation” of others, such as Margibi and Montserrado.

The median age of participants was 39 years (interquartile range, *IQR*: 32–48), and the majority was male (662/1120, 59.1%) (Table 1). Participants reported living with a median of five household members (*IQR*: 3–8). Approximately, 41% (454/1118) had a university education, while 22% reported having no formal education or primary education (249/1118). Health workers represented about 8% of the sample. Slightly over 12% indicated being unemployed, being retired, or not working due to school (122/1090). About 4% of the study sample reported having been told by a doctor that they had diabetes, and 13.5% reported a diagnosis of high blood pressure.

**Table 1:** Sociodemographic and health characteristics of the population of individuals who opted for COVID-19 vaccination in Liberia, by Ebola “near-miss” experience

**Characteristics**	**Overall (*n* = 1120)**	**Ebola near misses**^**a**^		
		**Yes (*n* = 69)**	**No (*n* = 1021)**	***p*-value**
**Age**				
**Median age (IQR)**	39 (32–48)	38 (34–48)	40 (32–48)	0.618
**Sex**				
**Male**	662/1120 (59.1)	45/69 (65.2)	599/1021 (58.7)	0.892
**Female**	458/1120 (40.9)	24/69 (34.8)	422/1021 (41.3)	
**Number of household members**				
**Median number of household members (IQR)**	5 (3–8)	6 (4–8)	5 (3–8)	0.368
**Highest education level completed**				
**No formal schooling**	144/1118 (12.9)	4/69 (13.0)	39/1019 (12.7)	0.815
**Primary school**	105/1118 (9.4)	8/69 (11.6)	94/1019 (9.2)	
**High school**	411/1118 (36.8)	27/69 (39.1)	373/1019 (36.6)	
**University**	458/1118 (41.0)	25/69 (36.2)	423/1019 (41.5)	
**Current occupation**^**b**^				
**Unemployed/retired/student**	254/1090 (23.3)	14/69 (20.3)	230/992 (23.2)	0.707
**Employed, health worker**	88/1090 (8.1)	4/69 (5.8)	81/992 (8.2)	
**Employed, not health worker**	748/1090 (68.6)	51/69 (73.9)	681/992 (68.6)	
**History of chronic conditions**				
**Diabetes**	46/1115 (4.1)	2/69 (2.9)	43/1016 (4.2)	0.999
**High blood pressure**	151/1118 (13.5)	19/69 (27.5)	128/1019 (12.6)	0.001
**Household or family member diagnosed with COVID-19**^**b**^				
**Yes**	35/1099 (3.2)	8/69 (11.6)	26/1007 (2.6)	< 0.001
**No**	1064/1099 (96.8)	61/69 (88.4)	981/1007 (97.4)	

Data presented as n/N (%) unless otherwise indicated. For each characteristic, *N* excludes those who responded “Don't remember” or “Don't know” or who refused to answer, unless otherwise indicated

^a^Excludes *n* = 27 participants who reported being “Unsure”

^b^Excludes *n* = 27 participants who reported “Don’t know”

Excluding those unsure, 6.3% of participants reported having a household or family member test positive for Ebola during the 2014–2016 outbreak (i.e., near misses) (69/1090). EVD near misses did not significantly differ from participants without close EVD experience in terms of age, sex, education level, household size, or employment status. However, they did report a significantly higher rate of hypertension (Table 1).

### Precautionary behaviors during EVD versus during COVID-19

**Fig. 2:**
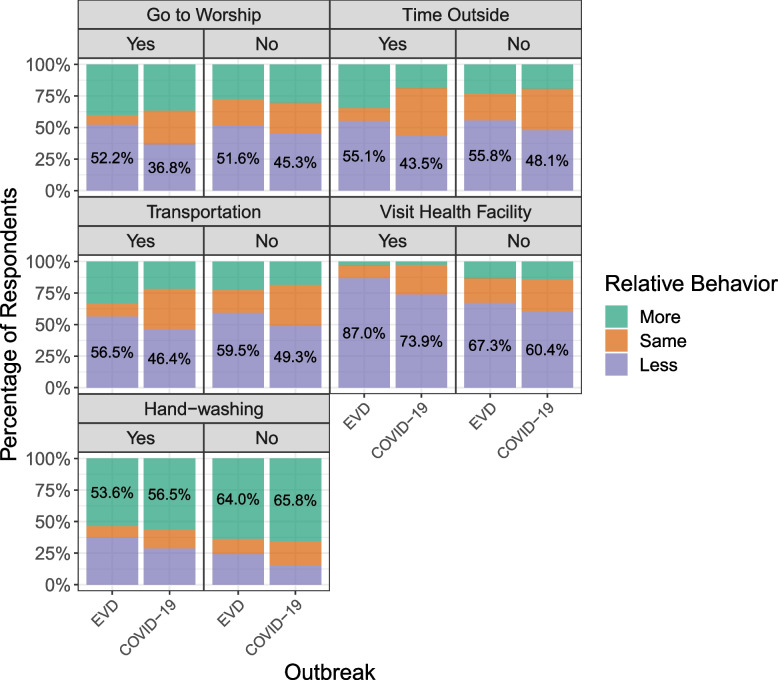
Relative behavior change for individual precautionary measures during Ebola versus during COVID-19. Behaviors were self-reported by participants as being undertaken at the same frequency, more often, or less often, relative to before the outbreak (Ebola or COVID-19). Results are further stratified by whether participants had close experience with disease in their families or households during the Ebola outbreak—that is, those who had a household member test positive for Ebola (yes) versus those who did not (no)

Adoption of precautionary behavior (i.e., less frequent practice of “risky” behaviors or more frequent practice of “preventative” measures) tended to be less during the COVID-19 outbreak in Liberia, relative to during the EVD outbreak (Table 2, Fig. 2). For instance, 51.3% of participants indicated less attendance at places of worship during Ebola than before Ebola, while 44.6% reported less attendance during COVID-19, relative to before the pandemic. This trend was consistent across all behaviors except for hand-washing. A slightly higher percentage of participants responded that they washed their hands more frequently during COVID-19 as compared to the reporting that they washed their hands more frequently during Ebola (65.1% versus 63.1%).

**Table 2:** Self-reported behavior change during the Ebola and COVID-19 outbreaks in Liberia, overall and by Ebola “near-miss” experience

**Frequency of behavior (relative to before the outbreak)**		**Overall (*n* = 1120)**	**Ebola near misses**^**a**^		
			**Yes (*n* = 69)**	**No (*n* = 1021)**	*p*-value**
**Going to places of worship**					
**During Ebola**	Less	571/1112 (51.3)	35/67 (52.2)	524/1015 (51.6)	0.010
	Same	230/1112 (20.7)	5/67 (7.5)	211/1015 (20.8)	
	More	311/1112 (28.0)	27/67 (40.3)	280/1015 (27.6)	
**During COVID-19**	Less	496/1112 (44.6)	25/68 (36.8)	459/1014 (45.3)	0.376
	Same	277/1112 (24.9)	18/68 (26.5)	245/1014 (24.2)	
	More	339/1112 (30.5)	25/68 (36.8)	310/1014 (30.6)	
**Spending time outside (i.e., leaving one’s house)**					
**During Ebola**	Less	620/1116 (55.6)	38/69 (55.1)	568/1018 (55.8)	0.026
	Same	229/1116 (20.5)	7/69 (10.1)	213/1018 (20.9)	
	More	267/1116 (23.9)	24/69 (34.8)	237/1018 (23.3)	
**During COVID-19**	Less	535/1120 (47.8)	30/69 (43.5)	491/1021 (48.1)	0.671
	Same	372/1120 (33.2)	26/69 (37.7)	333/1021 (32.6)	
	More	213/1120 (19.0)	13/69 (18.8)	197/1021 (19.3)	
**Using public transportation**					
**During Ebola**	Less	660/1120 (58.9)	39/69 (56.5)	607/1021 (59.5)	0.060
	Same	204/1120 (18.2)	7/69 (10.1)	184/1021 (18.0)	
	More	256/1120 (22.9)	23/69 (33.3)	230/1021 (22.5)	
**During COVID-19**	Less	548/1115 (49.1)	32/69 (46.4)	501/1017 (49.3)	0.797
	Same	361/1115 (32.4)	22/69 (31.9)	327/1017 (32.2)	
	More	206/1115 (18.5)	15/69 (21.7)	189/1017 (18.6)	
**Visiting a health facility**					
**During Ebola**	Less	753/1114 (67.6)	60/69 (87.0)	683/1015 (67.3)	0.001
	Same	215/1114 (19.3)	7/69 (10.1)	195/1015 (19.2)	
	More	146/1114 (13.1)	2/69 (2.9)	137/1015 (13.5)	
**During COVID-19**	Less	673/1116 (60.3)	51/69 (73.9)	614/1017 (60.4)	0.008
	Same	287/1116 (25.7)	16/69 (23.2)	257/1017 (25.3)	
	More	156/1116 (14.0)	2/69 (2.9)	146/1017 (14.4)	
**Washing hands**					
**During Ebola**	Less	280/1120 (25.0)	26/69 (37.7)	249/1021 (24.4)	0.047
	Same	133/1120 (11.9)	6/69 (8.7)	119/1021 (11.7)	
	More	707/1120 (63.1)	37/69 (53.6)	653/1021 (64.0)	
**During COVID-19**	Less	180/1119 (16.1)	20/69 (29.0)	158/1020 (15.5)	0.013
	Same	211/1119 (18.9)	10/69 (14.5)	191/1020 (18.7)	
	More	728/1119 (65.1)	39/69 (56.5)	671/1020 (65.8)	

Data presented as n/N (%) unless otherwise indicated. For each characteristic, *N* excludes those who responded “Don't remember” or “Don't know” or who refused to answer, unless otherwise indicated

^a^Excludes *n* = 27 participants who reported being ‘Unsure’ about whether any household or family member had Ebola

^**^Represents the results of the chi-squared test or Fisher’s exact test (when counts were < 5)

When considering behavior change by near-miss experience, it was observed that similar percentages of participants who reported having a close experience with Ebola and those who did not report near misses generally adopted precautionary behaviors. However, across outbreaks, the distribution of behavior change shifted for Ebola near misses, with higher percentages of near misses indicating more risky behavior adoption during Ebola than during COVID-19. For instance, among near misses, 34.8% of participants indicated spending time in public with higher frequency during the Ebola outbreak as compared to during non-outbreak times. However, during COVID-19, 18.8% spent more time outside as compared to before the pandemic. The within-group distribution shifts are analytically captured for behaviors where statistically significant differences were found between near misses and non-near misses for Ebola but not for COVID-19.

**Fig. 3:**
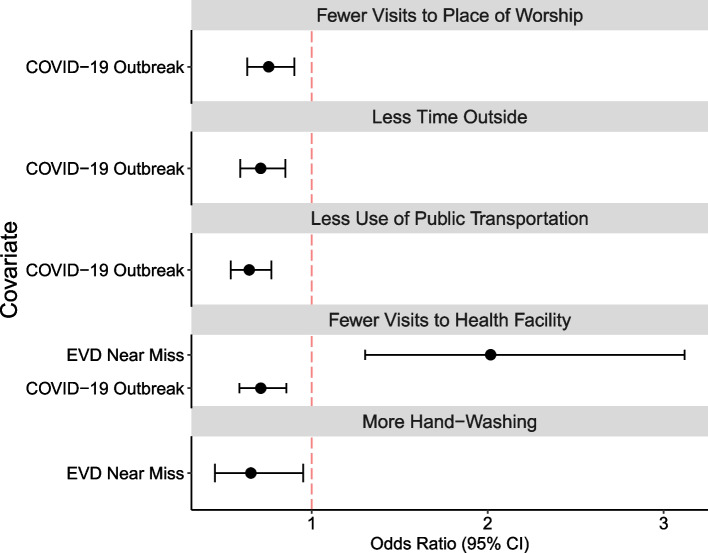
Results of multiple logistic regression analysis via GEEs for each precautionary behavior. The odds ratio of adoption of the behavior and the 95% confidence intervals are presented. Covariates represented in the figure reflect those that were selected in the model via QIC model selection as being most contributory to explaining the outcome behavior. The odds ratio for the COVID-19 outbreak represents the odds of behavior adoption during COVID-19 relative to during the Ebola outbreak. The odds ratio for the “near miss” represents the odds of behavior adoption by those with a close experience with disease during the Ebola outbreak relative to by those without a close experience

In quantifying the independent effects of outbreak and near-miss experience on precautionary behavior adoption, model selection for the outcome of individual precautionary behaviors tended to favor models that included outbreak as a covariate and that always excluded models with an interaction term for the outbreak and near-miss status (Additional file 2: Table S1 and Additional file 2: Fig S3). In other words, models that included an interaction term that would quantify how the adoption of precautionary behavior during successive outbreaks differed between Ebola near misses and those without close Ebola experiences did not explain the data better than models excluding the interaction term. Models with the interaction term had slightly higher QIC values than those excluding it. In contrast, after removing the interaction term, excluding the main effect of the outbreak often resulted in a higher QIC, suggesting that it should remain in the model. In general, the odds of adoption of precautionary behavior were significantly lower during the COVID-19 outbreak than during the EVD outbreak (Fig. 3). Participants were about 30% less likely to adopt precautionary behaviors with more frequency during COVID-19 than during Ebola.

Across behaviors, there were less consistent findings on how near-miss status (i.e., group variable) contributed to model fit. Near-miss status was found to be associated with behavior change around visits to health facilities and hand-washing, but it was not selected for inclusion in other models. The odds of making fewer visits to health facilities among EVD near misses were twice the odds among participants who had not experienced EVD among family or household members (*aOR*: 2.02, 95% *CI*: 1.30, 3.12). In contrast, the odds of more frequent hand-washing during COVID-19 among near misses were significantly less than the odds among those without close EVD experience (*aOR*: 0.66, 95% *CI*: 0.45, 0.95).

### COVID-19 vaccination, testing, and diagnosis

**Fig. 4:**
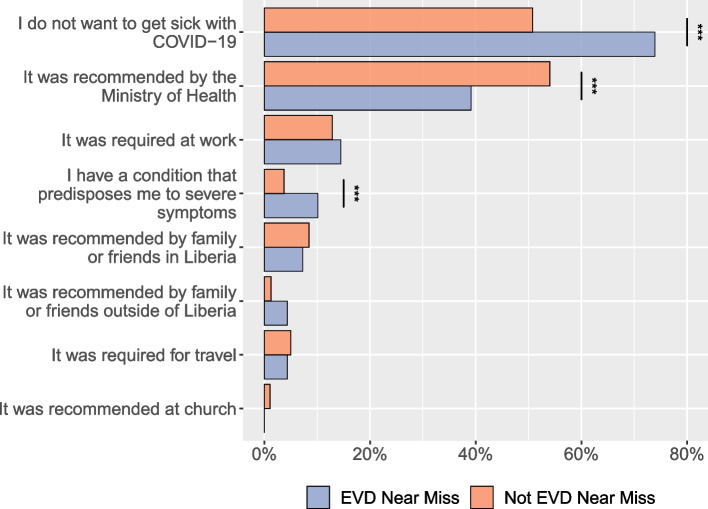
Reasons for getting the COVID-19 vaccine by EVD “near-miss” group. Participants indicated all factors that motivated them to get the COVID-19 vaccine, such that each percentage represents the percentage of participants in each “near-miss” group reporting a particular factor as part of their decision-making process. For differences between groups that were statistically significant at *p* < 0.05, they are noted by ***

Motivating factors behind the decision to take the COVID-19 vaccine differed between EVD near misses and those without close EVD experience. EVD near misses significantly more often reported concerns about personal health as the reason for COVID-19 vaccination (Fig. 4). Specifically, 74% of near misses (51/69), while 51% of the general population (517/1018), reported not wanting to get sick from COVID-19 as a driver in their decisions to get vaccinated (*p* < 0.001). EVD near misses also more often indicated that they sought the vaccine since they had conditions predisposing them to severe COVID-19 symptoms (10.1%, 7/69 for near misses versus 3.7%, 38/1018 for those who were not EVD near misses; *p* = 0.020). For those who did not report a near-miss experience, external guidance—specifically, recommendation by the Ministry of Health—was the most frequently identified reason for seeking COVID-19 vaccination. The percentage of the general population reporting recommendation by the MOH as its motivation (54.0%, 550/1018) was statistically significantly higher than the percentage of EVD near misses (39.1%, 550/1018) (*p* = 0.020).

Near misses were more likely to go for COVID-19 testing. About 13% reported going for testing after they received their first dose of the COVID-19 vaccine (13.0%, 9/69), while just over 8% of those without close EVD experiences reported going for testing (8.4%, 85/1018). However, the difference was not statistically significant. Out of the 84 participants who reported going for COVID-19 testing, only 3 were diagnosed as positive—none of whom were EVD near misses.

Just over 3% of the overall study sample reported having a household or family member diagnosed with COVID-19 during the pandemic (3.2%, 35/1099). A significantly higher percentage of those who reported near misses during the Ebola outbreak (11.6%, 8/69) reported having a household or family member diagnosed with COVID-19, as compared to the percentage of participants not reporting EVD near misses (2.6%, 26/1007) (*p* < 0.001) (Table 1). That is, the odds of a positive COVID test among household or family members of someone who reported having a household or family member test positive for EVD were 4.9 times those for someone who reported not having had a household or family member test positive for EVD.

### Future vaccine acceptance

**Fig. 5:**
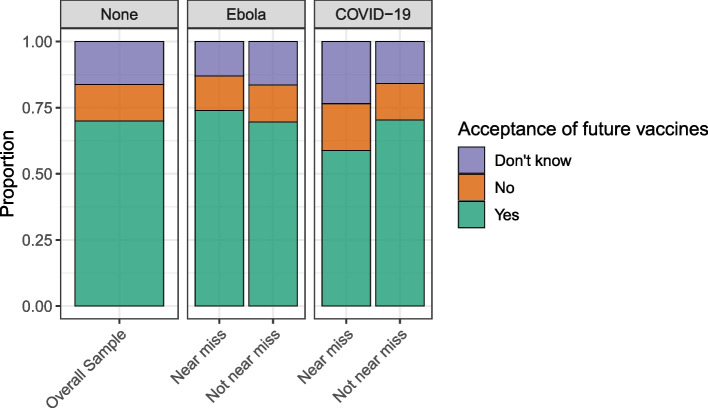
Willingness to accept Ebola vaccine if offered by local authorities. Participants reported whether they would be willing to accept a future prophylactic Ebola vaccine if it was available to them. The findings are presented for overall sample and by near miss status, both for EVD near misses and COVID-19 near misses

For the overall sample, 70% indicated they would accept an Ebola vaccine if it was available and offered (781/1116), while 16% were unsure (16.2%, 181/1116), and 14% indicated they would not accept it (13.8%, 154/1116) (Fig. 5). There was no significant difference in the distribution of acceptance between people with and those without close EVD experience or between people with and those without close COVID-19 experience (Table 3).

**Table 3:** Willingness to accept future vaccines, including prophylactic Ebola vaccines, overall and by “near-miss” status

**Future vaccination behavior**	**Overall (*n* = 1120)**	**Ebola near misses**			**COVID-19 near misses**		
		**Yes (*n* = 69)**	**No (*n* = 1021)**	***p*-value**	**Yes (*n* = 27)**	**No**	***p*-value**
**Willingness to accept future prophylactic Ebola vaccine**							
**Yes**	781 (70.0)	51 (73.9)	708 (69.6)	0.700	20 (58.8)	746 (70.3)	0.300
**No**	154 (13.8)	9 (13.0)	142 (14.0)		6 (17.6)	147 (13.9)	
**Undecided**	181 (16.2)	9 (13.0)	167 (16.4)		8 (23.5)	168 (15.8)	

Data presented as n/N (%) unless otherwise indicated. For each characteristic, *N* excludes those who responded “Don't remember” or “Don't know” or who refused to answer unless otherwise indicated

### Perceptions around the Ebola and COVID-19 experiences in Liberia

In-depth interview participants primarily expressed negative feelings when discussing the two outbreaks. Both Ebola and COVID-19 tended to be perceived as deadly.

### More proximal and personal experiences with Ebola than with COVID-19

Although COVID-19 was often considered deadly, participants reflected on how populations outside of Liberia mostly felt its potential to kill, while the possibility of another Ebola epidemic still stokes fear locally. The “deadliness” of Ebola were explained in the context of participants’ personal experiences or experiences of people around them.

COVID… affected everybody unlike Ebola that was concentrated in a particular region or particular country, COVID affected the whole world. However, whenever I hear the word Ebola, I feel terribly sad because on average, Ebola was riskier than Covid 19. I knew some people that died from the Ebola virus. Up to now I still feel sad when I hear the word Ebola.

I lost some family and friends during the Ebola time so it is still affecting me up to now… and my sister because she lost her husband during the Ebola time and didn’t have the time to see him nor bury him. I still get frightened up to now when I hear Ebola.

None of my family [died from Ebola] but some of my friends. Like, I had one of my friends who the Ebola took her mother, her little brother, the brother’s wife…almost five persons in the house. Ebola killed them.

Almost all interviewees compared the two outbreaks and often emphasized that the Ebola experience had been worse than COVID-19 or that fear from the Ebola experience affected their perceptions of COVID-19.

When I heard about COVID, I said it was the same thing that happened during Ebola coming to happen again. I was afraid that one of my family members could go, or even me, since Ebola missed us.

I will not say [COVID] scares me as equal to Ebola, you know the trauma I went through at that time… so I mean, yes, [COVID] is a disease that I wish that nobody catches, but it’s the world that we are living in, we just supposed to accept it as it is.

### Lessons learned during the EVD outbreak minimized impact of COVID-19

Interview participants identified protective behaviors and often suggested that they were learned during Ebola. One participant even suggested that the time to adopt precautionary behaviors during Ebola, given their general absence before, contributed to the severity of the outbreak in Liberia.

We still need to do those basic things like washing of hands or [use of] sanitizer, avoiding public gathering at times. Because it is with these things that we the country will still need to do in future if there will be another outbreak and also it was these major things that we didn’t do during the Ebola time that caused a lot of deaths.

### Lack of a vaccine during the EVD outbreak and common misconceptions around COVID-19 vaccine in the absence of past experience

While the EVD experience was noted as preparing systems and individuals for how to respond to COVID-19 in several ways, it did not provide experience with mass vaccination. Multiple interviewees noted that the lack of a vaccine during the Ebola outbreak had rendered Liberians more vulnerable. Some indicated that this perception motivated their COVID-19 vaccination behavior.

[I took the COVID-19 vaccine] because you know due to the Ebola crisis that killed a lot of people because we didn’t have vaccines.

When I hear COVID-19, I can just link that same kind of fear from Ebola to COVID. So I was encouraged to take the right procedures to protect myself and my family against that kind of sickness and when the COVID vaccine came, I was so encouraged to be part of it.

In other ways, the lack of a vaccine for population-level use during the EVD outbreak in West Africa—despite efforts to develop and test one—provided no prior experience with accepting new vaccines in the midst of an outbreak. Misconceptions and misinformation about the COVID-19 vaccine were common across social media platforms and in casual discourse. Respondents stated people were afraid of dying from COVID-19, but they also were afraid to take the vaccines.

Yeh….Yeh…I took the vaccine. Ehnn…since I took that vaccine, the only fear that was in me was the Liberian people saying….when you take the vaccine two years after you will die. That was the fear that was in me, but when I took the vaccine, nothing bad happen, no sickness even give me hard time. I naaaa feel no effect on my body, no pain sehhh on my body to say my arm heavy, nothing like that. Other people were saying when they took the vaccine their arm get swollen, they were feeling cold…Nothing like that happen to me.

I just wanted to take the COVID-19 vaccine, because other people say it will kill you. Ayyyyy will do this one to you, so I say let it kill me. Let me go and take the COVID-19 vaccine. Daaaa the thing business I went there to take the vaccine. People say when they take it their head can hurt them, their head can get swollen but I say I will go take it and daaaa how I went out to take the vaccine.

## Discussion

Liberia’s experience during COVID-19 was inevitably compared to the experience with Ebola 5 years earlier. This occurred both at the systems level to suggest how recent history with a widespread, high-morbidity outbreak could better prepare Liberia for managing COVID-19 and also at the individual level to consider how perceived risk and willingness to change behavior during COVID-19 may have been informed by perceived risk and behavior change during Ebola. Our study suggests that Liberians often compared the successive outbreaks in terms of intensity, proximity, and preparedness. In addition to considering COVID-19 less severe than Ebola in terms of its local impact, they perceived higher preparedness for COVID-19 due to the population’s experiences with behavior change during the EVD outbreak. Yet, despite this recently developed, preexisting awareness on personal protective measures, degree of adoption during COVID-19 seemed less than during the Ebola outbreak per the study’s self-reported phone survey data. It had been hypothesized that individual-level, proximal experiences with Ebola (“near misses”) may have influenced differential changes in behavior during COVID-19 compared to changes in the absence of close experiences with Ebola. In general, however, near-miss Ebola experiences were not associated with significant differences in precautionary behavior adoption during the pandemic.

While the study did not provide evidence that near-miss Ebola experiences moderated differential uptake of precautionary behavior during COVID-19, it was observed that the prevalence of near misses reported in the study sample was higher than may have been expected at the population level. At the national level, it could be estimated that approximately (and conservatively) 1.1% of Liberians would have had a household member who had Ebola, per the reported 10,678 confirmed, probable, and suspected EVD cases [32], the estimated 2016 population size, and the average 4.6 persons per household [47]. However, in our study sample of adult Liberians vaccinated against COVID-19, 6.3% (69/1021) of participants reported having a family or household member affected by Ebola. The observed “overrepresentation” of closely affected individuals in this study of vaccinated adults could be because the near-miss experience led to high vaccine acceptance despite the adoption of other precautionary behavioral measures not being different among near misses relative to non-near misses. This is consistent with the finding that near misses were motivated by personal health-related reasons to pursue COVID-19 vaccination, while the general population expressed significantly more influence from external influences, namely the Ministry of Health. Of note, it is not expected that vaccination moderated the impact of near-miss experiences on other personal behavior changes since the delay in the rollout of vaccination in Liberia most likely meant that the non-pharmaceutical protective behaviors being reported were during the first waves when government policies around lockdowns, curfews, and mask wearing were in place.

The current study investigated individual-level behavior adoption during outbreaks. It sought to gain insight into perceptions around behavior decisions and around systems-level preparedness for recommending and supporting such behavior change. It is important to acknowledge that community-level experiences and decisions likely had a role—even if not directly measured—in the findings. Community-level efforts played a key role in motivating and supporting behavior change during the Ebola outbreak in Liberia and Sierra Leone [48, 49]. Previous work demonstrated how risk communication and community engagement (RCCE) strategies may have mitigated some of the needs-related barriers to adopting transmission-reducing behaviors [30]. The role of community-based initiatives and RCCE in moderating the impact of personal experience during past outbreaks on individual (and collective) decisions during a new outbreak warrants consideration as part of strengthening post-COVID preparedness for the next public health threat.

This study was embedded within an overarching study to characterize the population that sought out COVID-19 vaccination once it was available in Liberia and to understand their experiences with vaccination. While this sample is not representative of the entire Liberian population, it was assumed to reflect people who had recently engaged with the health system and who could provide insights that could be leveraged to try and better reach the unvaccinated population. Moreover, the use of vaccinated adults allowed for access to a database of contact numbers for connecting with potential participants via a phone survey, a methodology that was important for averting further transmission that could result if study teams engaged in field-based data collection. Although all participants had been vaccinated at the time of the survey, the findings suggest that their motivations for getting vaccinated varied, with people who had had close experiences with Ebola significantly more often reported concerns about personal health as the reason for COVID-19 vaccination and those without close experiences reporting external influences, like recommendations from MOH, as driving their decisions. Such findings support multipronged efforts to affect vaccination intentions [36, 50, 51] while also emphasizing the need for context-specific understanding of risk perception in low-income settings with prevalent experience with infectious disease outbreaks [31]. Sensitivity to motivations behind vaccine acceptance, as well as to the effects of practical and other factors influencing vaccine uptake, has been conceptualized [52] and found to be effective in predicting and understanding vaccination behavior [53, 54]. As public health researchers and practitioners anticipate vaccine needs for future outbreaks, socio-behavioral frameworks of vaccine acceptance will be important to generate demand, particularly in groups that may be prioritized in the event of limited supply or other logistical constraints [55].

### Limitations

While necessitated, in part, by the need to protect data collectors and participants from interactions that could lead to transmission, the use of a phone survey likely led to selection bias such that the sample cannot be assumed to be representative of all vaccinated adults nationally. Several attempts were made to reach potential participants to try to reach those who may have been affected by inconsistent cellular network at certain times of the day or in certain locations in Liberia. However, the overrepresentation of certain counties in the final sample reflects that potential participants in more remote counties were less accessible. In particular, the coverage of the phone survey relative to the sampling pool of vaccinated Liberian adults rendered the results less generalizable to rural settings with low cellular network coverage; these are settings where the behaviors under investigation may have been less relevant due to limited access to public transport networks, places of worship, and health facilities, for instance. Likewise, digitization of the paper data also introduced bias, as some counties had entered less paper-based data than others at the time of sampling. While the project was intentionally delayed to allow for entry from the paper ledgers, the process was ongoing during sampling due to a significant backlog. The team balanced the need for timely results with the importance of a representative sample. We aimed to address potential biases due to sampling processes and participation rates at the analysis stage by adjusting for sociodemographic factors in the models. In addition, as with many survey studies, our results may be impacted by recall and social desirability biases due to the use of self-reported behavior and self-reported close experiences with Ebola.

Moreover, the framing of the questions on relative behavior change did not capture the possibility that people had maintained higher adoption of precautionary behaviors in the inter-epidemic period, with the survey question did capture absolute coverage of the behaviors. However, despite such limitations that may affect the generalizability of the results, the findings offer insight into how the Ebola experience may have impacted decision-making around the adoption of preventative measures during COVID-19 and provide a framework for including past outbreak experience in predicting and understanding responses to future outbreaks. Future work can leverage these findings to inform interventions and how to plan for their evaluation during subsequent outbreaks, as well as more intentionally investigate experiences in less accessible areas where alternative behavioral strategies may be more relevant.

## Conclusions

In Liberia, reported precautionary behavior adoption in response to COVID-19 was significantly less than precautionary behavior change in response to Ebola. People with direct Ebola experience were also more likely to have reported experiencing COVID-19 among family or household members. However, they were similar in their adoption of precautionary behavior during COVID-19 as compared to people without close Ebola experience. The findings here reflect how successive outbreaks with widespread impact could affect decision-making around behavior change, such as due to comparisons in the observed and experienced severity of the disease. This has implications for local response and global health security. It is recommended that individuals within the population and authorities within health institutions learn from past outbreaks but also recognize differences in epidemiology across outbreaks and be sensitive to the complexities of multilevel influences in individual risk assessment. Development of contextually relevant innovations based on this learning should be prioritized for investment during inter-epidemic times.

## Supplementary Information

**Additional file 1.** STROBE checklist.

**Additional file 2: Figure S1.** Proposed proportional sampling and difference between the proposed and actual sampling in terms of the proportion of the sample from each county. Sampling of potential participants was done from the 15 counties of Liberia according to the proportion of the population receiving a vaccine at the time of the study and per data reported by the Expanded Programme on Immunization. The ultimate sample of phone survey participants differed from the sample taken from the full database of vaccinated adults based on those who were reachable via phone and who consented to participate. The final sample under-represented vaccinated individuals in Grand Bassa, Lofa, and Maryland Counties, while over-representing vaccinated individuals in Margibi and Montserrado Counties, for instance. **Table S1.** Results of GEE Model Selection. Phone Survey Data Collection Tool. In-Depth Interview Data Collection Tool.

## Data Availability

All data collection tools are available in Additional file 1. Survey data elements used for this substudy and de-identified transcripts from the in-depth interview questions are available from the corresponding author upon request, per the data availability provisions in the approved IRB protocol.
