# Supplementary material for: Unraveling the effects of the Ebola experience on behavior choices during COVID-19 in Liberia: a mixed-methods study across successive outbreaks
Source: BMC Glob Public Health. 2024 Apr 1;2:22. doi: 10.1186/s44263-024-00054-5 (PMC11622887; doi:10.1186/s44263-024-00054-5)
Supplement: Supplementary file 2 — Additional file 2: Figure S1. Proposed proportional sampling and difference between the proposed and actual sampling in terms of the proportion of the sample from each county. Sampling of potential participants was done from the 15 counties of Liberia according to the proportion of the population receiving a vaccine at the time of the study and per data reported by the Expanded Programme on Immunization. The ultimate sample of phone survey participants differed from the sample taken from the full database of vaccinated adults based on those who were reachable via phone and who consented to participate. The final sample under-represented vaccinated individuals in Grand Bassa, Lofa, and Maryland Counties, while over-representing vaccinated individuals in Margibi and Montserrado Counties, for instance. Table S1. Results of GEE Model Selection. Phone Survey Data Collection Tool. In-Depth Interview Data Collection Tool. [file 44263_2024_54_MOESM2_ESM.docx]

Additional file 2

Unraveling the effects of the Ebola experience on behaviour choices during COVID-19 in Liberia: A mixed methods study of ‘near misses’ and risk perception across successive outbreaks

Laura A. Skrip, Malcom B. Weller, Sheikh Dukuly, Neima Candy, Wahdae-Mai Harmon-Gray, Adolphus Clarke, Bernice T. Dahn

List of supplementary contents:

- Figure S1. Proposed proportional sampling and difference between the proposed and actual sampling in terms of the proportion of the sample from each county.
- Table S1. Results of GEE Model Selection
- Phone Survey Data Collection Tool
- In-Depth Interview Data Collection Tool

*
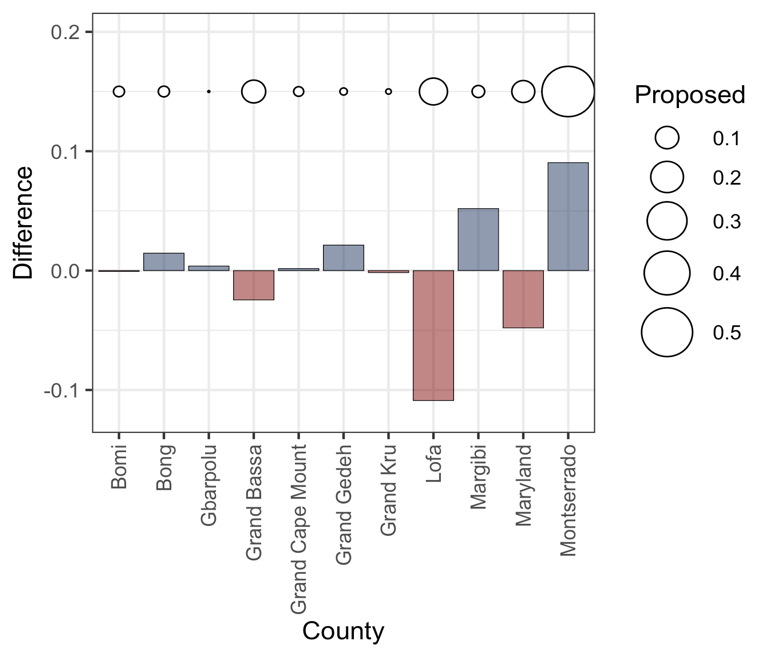
*

*Figure S1. Proposed proportional sampling and difference between the proposed and actual sampling in terms of the proportion of the sample from each county. Sampling of potential participants was done from the 15 counties of Liberia according to the proportion of the population receiving a vaccine at the time of the study and per data reported by the Expanded Programme on Immunization. The ultimate sample of phone survey participants differed from the sample taken from the full database of vaccinated adults based on those who were reachable via phone and who consented to participate. The final sample under-represented vaccinated individuals in Grand Bassa, Lofa, and Maryland Counties, while over-representing vaccinated individuals in Margibi and Montserrado Counties, for instance.*

*Table S1. Results of GEE Model Selection*

| **Precautionary Behavior** | **QIC** | **aOR (95% CI)** | **P-value** |  |  |  |
| --- | --- | --- | --- | --- | --- | --- |
| **Less Frequent Visits to Places of Worship** | | | |  |  |  |
| Full Model* | 2757 |  |  |  |  |  |
| Interaction Term Removed | 2757 |  |  |  |  |  |
| **+ Group Main Effect Removed** | **2756** |  |  |  |  |  |
| + Outbreak Main Effect Removed | 2764 |  |  |  |  |  |
| + Both Group and Outbreak Main Effects Removed | 2764 |  |  |  |  |  |
| Selected Model: Group Main Effect Removed |  |  |  |  |  |  |
| Group: EVD Near Miss ** |  |  |  |  |  |  |
| Outbreak: COVID-19 ** |  | 0.76 (0.63, 0.90) | 0.002 |  |  |  |
| Interaction: Group x Outbreak |  |  |  |  |  |  |
| **Less Time Outside the Home (in Public Spaces)** |  |  |  |  |  |  |
| Full Model* | 2704 |  |  |  |  |  |
| Interaction Term Removed | 2703 |  |  |  |  |  |
| **+ Group Main Effect Removed** | **2702** |  |  |  |  |  |
| + Outbreak Main Effect Removed | 2715 |  |  |  |  |  |
| + Both Group and Outbreak Main Effects Removed | 2714 |  |  |  |  |  |
| Selected Model: Group Main Effect Removed |  |  |  |  |  |  |
| Group: EVD Near Miss |  |  |  |  |  |  |
| Outbreak: COVID-19 |  | 0.71 (0.59, 0.85) | <0.001 |  |  |  |
| Interaction: Group x Outbreak |  |  |  |  |  |  |
| **Less Frequent Use of Public Transport** |  |  |  |  |  |  |
| Full Model* | 2716 |  |  |  |  |  |
| Interaction Term Removed | 2714 |  |  |  |  |  |
| **+ Group Main Effect Removed** | **2712** |  |  |  |  |  |
| + Outbreak Main Effect Removed | 2735 |  |  |  |  |  |
| + Both Group and Outbreak Main Effects Removed | 2733 |  |  |  |  |  |
| Selected Model: Group Main Effect Removed |  |  |  |  |  |  |
| Group: EVD Near Miss |  |  |  |  |  |  |
| Outbreak: COVID-19 |  | 0.65 (0.54, 0.77) | <0.001 |  |  |  |
| Interaction: Group x Outbreak |  |  |  |  |  |  |
| **Less Frequent Visits to Health Facility** |  |  |  |  |  |  |
| Full Model* | 2562 |  |  |  |  |  |
| **Interaction Term Removed** | **2561** |  |  |  |  |  |
| + Group Main Effect Removed | 2570 |  |  |  |  |  |
| + Outbreak Main Effect Removed | 2572 |  |  |  |  |  |
| + Both Group and Outbreak Main Effects Removed | 2581 |  |  |  |  |  |
| Selected Model: Interaction Term Removed |  |  |  |  |  |  |
| Group: EVD Near Miss |  | 2.02 (1.30, 3.12) | 0.002 |  |  |  |
| Outbreak: COVID-19 |  | 0.71 (0.59, 0.86) | <0.001 |  |  |  |
| Interaction: Group x Outbreak |  |  |  |  |  |  |
| **More Frequent Handwashing** |  |  |  |  |  |  |
| Full Model* | 2614 |  |  |  |  |  |
| Interaction Term Removed | 2612 |  |  |  |  |  |
| + Group Main Effect Removed | 2615 |  |  |  |  |  |
| **+ Outbreak Main Effect Removed** | **2611** |  |  |  |  |  |
| + Both Group and Outbreak Main Effects Removed | 2614 |  |  |  |  |  |
| Selected Model: Outbreak Main Effect Removed |  |  |  |  |  |  |
| Group: EVD Near Miss |  | 0.66 (0.45, 0.95) | 0.027 |  |  |  |
| Outbreak: |  |  |  |  |  |  |
| Interaction: Group x Outbreak |  |  |  |  |  |  |
| * Full Model: Behavior ~ **(Outbreak*Group) + Outbreak + Group + Sex + Age + Education + Region**  ****** For the Group variable, being an EVD near miss was compared to the reference level of not being an EVD near miss; for the Outbreak variable, the timepoint of the COVID-19 outbreak was compared to that of the EVD outbreak. | | | |  |  |  |

Phone Survey Data Collection Tool

Assessing Social, Demographic, and Clinical Drivers of COVID-19 Vaccination Behavior in post-Ebola Liberia

**I am interested in what motivated you to go for the vaccine.**

A.1. Why did you choose to go for the vaccine?

Please select all that the participant suggests. If the answer is not listed, choose other. Then type the reason in the next question.

- Recommended by family or friends in Liberia
- Recommended by family or friends outside of Liberia
- Recommended at church
- Required for travel
- Required for work
- Based on information from MOH
- Since I have a condition that predisposes me to severe symptoms
- Since I do not want to get sick with COVID-19
- Other

A.1.a. If you chose Other reason, please specify here.

**Now, I am going to ask you some questions about what your experience was when you went for the vaccine.**

B.1.a. When did you first go for the vaccine? Which month?

Enter 99 if participant does not remember.

B.1.b. When did you first go for the vaccine? Which day?

Enter 99 if participant does not remember.

B.1.c. When did you first go for the vaccine? Which year?

Enter 99 if participant does not remember.

B.2. Where did you go to get your first dose of the COVID-19 vaccine?

Please select all that the participant suggests. If the answer is not listed, choose other. Then type the reason in the next question.

- Health facility
- Church
- Pop-up campaign in community
- Other

B.2.a. If Other indicated, please specify here.

B.3. About how many minutes did you wait in line to get the first dose of the vaccine? (Enter in whole number)

B.4. Did you receive a vaccine card at the vaccination site?

- Yes
- No

B.5. Would you recommend that your friends go to the same vaccination site or try a different one?

- Same
- Different
- I wouldn’t recommend that my friends go for vaccination

B.6. When you first got the vaccine, during the same day or the next day, did you feel any sickness or side effects?

- Yes
- No
- Don’t remember

**I am going to list some commonly reported side effects of the vaccine. These reflect a list that the World Health Organization has indicated as common, mild-to-moderate side effects. Please state yes or no to let me know if you experienced them.**

B.7.a. Fever

- Yes
- No
- Don’t remember

B.7.b. Fatigue

- Yes
- No
- Don’t remember

B.7.c. Headache

- Yes
- No
- Don’t remember

B.7.d. Muscle Pain

- Yes
- No
- Don’t remember

B.7.e. Chills

- Yes
- No
- Don’t remember

B.7.f. Diarrhea

- Yes
- No
- Don’t remember

B.7.g. Pain at the injection site

- Yes
- No
- Don’t remember

B.7.h. Other symptoms? (Please list)

B.8. [If YES to #6] Overall, how bad were the symptoms?

- Very severe
- Somewhat severe
- Not really severe
- Not severe at all

B.9. [If YES to #6] Did they make you miss work or school or other activities due to the side effects?

- Yes
- No
- Don’t remember

B.10. Did you experience any symptoms of respiratory infection like coughing, sneezing, or difficulty breathing since you were first vaccinated?

- Yes
- No
- Don’t remember

B.11. [If YES to #10] How long after the vaccine did you get sick? (Days/Weeks/Months)

B.12. [If YES to #10] How many days did you feel that way? Enter 999 if unknown

B.13. Were you tested for COVID-19 since you were vaccinated with the first dose?

- Yes
- No

B.14. [If YES to #13] What was the result?

- Positive
- Negative

B.15. [If received only a single dose of AstraZeneca at time of questionnaire] How likely are you to go back for the second dose?

- Not at all likely
- A little likely
- Very likely
- Certainly likely
- Not applicable

B.16. Have you heard of a booster dose?

- Yes
- No
- Don’t know

B.17. How likely are you to go back for a booster?

- Not at all likely
- A little likely
- Very likely
- Certainly likely
- Don’t know what a booster is

B.18. Do you feel safer since you got vaccinated?

- No
- Yes, a little
- Yes, a lot

B.19. We are wondering if you interested in other vaccines now that you got the COVID vaccine. If an Ebola vaccine were offered, would you accept it?

- Yes
- No
- Don’t know

There are a few more sections to this survey. Thanks for your answers so far. I will now ask you about your behaviors during the outbreaks, both COVID-19 and Ebola Outbreaks

C.1. Did any of your household members of family members get a positive test for COVID-19 during the pandemic?

- Yes
- No
- Don’t know

C.2. During the COVID-19 outbreak, have you changed your behavior in the following ways? Please state whether you did each activity the same amount, more, or less than when COVID-19 was not in Liberia.

C.2.a. I use public transport…

- More
- Less
- Same

C.2.b. I wash my hands…

- More
- Less
- Same

C.2.c. I leave my house…

- More
- Less
- Same

C.2.d. I go to worship (church or mosque)…

- More
- Less
- Same

C.2.e. I go to the health facility…

- More
- Less
- Same

C.2.f. I travel to other counties or countries…

- More
- Less
- Same

C.3. Yesterday, how many minutes did you have the facemask on? Please only include time that it was covering your nose (not under your chin)?

C.4. Have you ever worn a facemask over you nose in the following places?

C.4.A. Public Transport

- Yes
- No

C.4.B. Market

- Yes
- No

C.4.C. Workplace

- Yes
- No

C.4.D. Home

- Yes
- No

C.4.E. Personal vehicles

- Yes
- No

C.4.F. If you wore a facemask anywhere else, please indicate it.

C.5. Did any of your household members or family members get Ebola during the outbreak in 2014-2015?

- Yes
- No
- Unsure

C.6. During the Ebola outbreak, what did you do differently? Please state whether you did each activity the same amount, more, or less than when Ebola was not in Liberia.

C.6.a. I used public transport…

- More
- Less
- Same

C.6.b. I washed my hands…

- More
- Less
- Same

C.6.c. I left my house…

- More
- Less
- Same

C.6.d. I went to worship (church or mosque)…

- More
- Less
- Same

C.6.e. I went to the health facility…

- More
- Less
- Same

C.6.f. I traveled to other counties or countries…

- More
- Less
- Same

**Now, I would like to know about any health conditions you may be experiencing now or have experienced in the past. These are not because of COVID-19 but just other health conditions.**

D.1. Have you ever been told by a doctor that you have diabetes or sugar?

- Yes
- No

D.2. Have you ever been told by a doctor that you have high blood pressure?

- Yes
- No

D.3. Have you ever been told by a doctor that you have sickle cell disease?

- Yes
- No

D.4. Are you currently receiving treatment for tuberculosis?

- Yes
- No

D.5. Are you currently receiving treatment for HIV?

- Yes
- No

Lastly, we will ask some demographic questions.

E.1. Please tell me how old you are? (In whole number years)

E.2. In which community do you stay?

E.3. Which district is that community located in?

E.4. What is your occupation?

- Unemployed
- Selling/Business
- Farming
- Motorbike Rider
- Teacher
- Student
- Other

E.4.a. If Other occupation selected, please describe.

E.5. How many other people stay in the same household as you? (in whole number)

E.6. How many of the other people in your household are adults, 18 years or older?

E.7. What is the highest level of education you have completed?

- No school
- Primary School
- High School
- University
- Vocational School

Thank you for making time to talk. Were you nervous about participating in this survey over the phone?

- Yes
- No

Would you participate in future phone surveys like this?

- Yes
- No

In-Depth Interview Data Collection Tool

Assessing Social, Demographic, and Clinical Drivers of COVID-19 Vaccination Behavior in post-Ebola Liberia

1. Please tell me about why you chose to get vaccinated?

2. What was your vaccination experience like? Like where did you go and how were you treated there?

3. Is there a reason you chose the vaccine you did? I have noted that you took {SPECIFY REGIMEN]

4. Please tell me about what other precautions you have taken to protect yourself against COVID-19?

5. Were you ever hesitant to get the vaccine? If so, what were your concerns and how did you change your mind?

6. Would you recommend to your friends that they get vaccinated? If so, what would you want them to know and understand about the vaccine and about the process of getting vaccinated?

7. Tell me about how you feel when you hear the word Ebola. Why do you feel that way?

8. Tell me about how you feel when you hear the word COVID-19. Why do you feel that way?

9. Do you think that Liberia could do anything differently to protect itself from future outbreaks of disease? What would you like to see?

10. Lastly, how did you feel about participating in the phone survey? We recognize it is not common in Liberia and may have been strange to some people.
